# Feasibility of Remote Performance Assessment Using the Free Research Executive Evaluation Test Battery in Adolescents

**DOI:** 10.3389/fpsyg.2021.723063

**Published:** 2021-10-14

**Authors:** Isis Angelica Segura, Sabine Pompéia

**Affiliations:** Departamento de Psicobiologia, Universidade Federal de São Paulo, São Paulo, Brazil

**Keywords:** adolescents, executive functions, COVID-19, online testing, updating, inhibition, shifting

## Abstract

Lockdowns and other preventive measures taken to curb the spread of diseases such as COVID-19 have restricted the use of face-to-face cognitive assessment. Remote testing may be an alternative, but it should first be shown to be comparable to in-person assessment before being used more widely, during and after the pandemic. Our aim was to evaluate the suitability of online, examiner-mediated administration of an open-access battery of executive function tests (the Free Research Executive Evaluation battery, or FREE) that can be adapted considering various characteristics of diverse populations and therefore used worldwide. A total of 96 9–15-year olds (42 girls) were tested, half of whom online through video calls mediated by an examiner. Their performance was compared to that of the other 48 individuals tested face-to-face, who were matched against the online-tested participants for age, pubertal status, sex, and parental schooling. The battery consists of two tests of the following executive domains: Updating (2-Back and Number Memory tests), Inhibition (Stroop Victoria and Stroop Happy-Sad), and Switching (Color Shape and Category Switch). Answers were vocal and self-paced, and the examiner recorded accuracy and time taken to complete in-person and online tasks. Only free software is needed for the assessment. Executive measures obtained from the tasks did not differ statistically between online and in-person tested participants and effects sizes of group effects were small, thus showing that the FREE test battery holds promise for online cognitive assessment, pending confirmation in different samples and further validation studies.

## Introduction

In the context of the COVID-19 pandemic’s social distancing, researchers interested in cognition have looked at the feasibility of remote cognitive testing. Perhaps surprisingly, there is a substantial body of evidence from the last two decades showing that online cognitive assessment may be equivalent to lab-based, face-to-face testing (Krantz and Dalal, 2000; McGraw et al., 2000; Nosek et al., 2002; Temple et al., 2010; Soto et al., 2011; Germine et al., 2012; Cullum et al., 2014) in elderly (e.g., Geddes et al., 2020), adult (e.g., Kirkwood et al., 2000), and pediatric populations (e.g., Hodge et al., 2019; Worhach et al., 2021). Although the validity and reliability of remote assessment has been questioned due to difficulties in controlling stimuli presentation and measuring response, both in terms of accuracy and reaction times (Germine et al., 2012), remote testing has unprecedent advantages that make it worth pursuing. As long as testees have internet access, these advantages include: (1) less travel time and expense, as well as lower implementation costs (Reips, 2000); (2) the possibility of reaching more diverse and less accessible samples, such as those from remote countries or areas, and/or patients with clinical conditions such as reduced mobility and/or higher vulnerability to diseases like COVID-19; and (3) testing people in familiar settings (their homes) instead of unknown locations, which has been shown to improve performance on some types of tasks (Sucksmith et al., 2013).

These advantages extend to testing BAME (Black, Asian and other non-white minority ethnic backgrounds) and non-WEIRD (Western Educated Industrialized Rich and Democratic) populations with internet access. These minorities or minoritized individuals have been under-represented in the cognitive literature in general, despite being more representative of humanity as a whole (see Henrich, 2010; Rad et al., 2018). Cultural, socioeconomic and ethnic differences affect not only the cognitive processes one might expect (e.g., social cognition and moral judgement) but also abilities such as visual perception, memory, categorization, attention, and executive functions (EF) (Henrich, 2010; Kelkar, 2013; Hackman and Gallop, 2015). EF is an umbrella-term for top-down cognitive functions that regulate an individual’s behavior and emotions in order to achieve goals that are in peoples’ minds (in their working memory) (Baggetta and Alexander, 2016; Friedman and Miyake, 2017). These behavioral self-regulation abilities have been found to be affected by many factors related to developing nations’ poor, minority and minoritized groups (Moffitt et al., 2011), which have been hit harder by the pandemic, likely a longer-lasting threat for them due to a host of environmental factors (see Silva and Ribeiro, 2021).

In this scenario, remote EF cognitive testing must ensure health and safety of testees and examiners and also allow administration of cognitive tests that are adaptable to different cultural and socioeconomic contexts so as to more reliably capture the cognitive constructs under investigation (Fernández and Abe, 2018). Bearing this in mind, we investigated the adequacy of remote EF assessment mediated by examiners using a test battery designed to be adaptable to different contexts and populations (FREE: Free Research Executive Function Evaluation; Zanini, 2021).

The FREE test battery includes tasks that measure three types of executive functions that are interrelated, yet separable, based on a theoretical framework called the Unity and Diversity of Executive Functions (see Friedman and Miyake, 2017). These types of EF are inhibition of automatic responses, shifting between tasks, and updating information held in working memory. Importantly, these tasks were adapted for affordable testing by researchers using basic equipment. Task presentation and scoring are not automated. Testees themselves regulate the speed at which they can do tasks and respond vocally while the examiner measures accuracy and time taken to complete blocks of trials instead of each individual trial. This is important because many studies have shown that limiting exposure and response times and requiring key presses for verbal answers can negatively influence measurements of EF in samples that include participants with different characteristics, such as various ages, and who are from diverse backgrounds (see Zanini, 2021). This test battery may therefore be administered remotely and be moderated by an online examiner, using screen sharing services that may be downloaded and used by examiners and testees free of charge without any special hardware, software downloads or plug-ins.

Here, the performance of adolescents tested online was compared with that of adolescents tested face-to-face in their own schools. Investigating EF is especially important during this phase of life because these cognitive skills develop during this period becoming differentiable in three distinct domains (see Lee et al., 2013), so factors that affect the environment and health at this age can potentially impact the development of EF performance, which influences a wide range of outcomes such as physical and mental well-being, academic and financial success, criminal and addictive behavior (see Moffitt et al., 2011). Hence, EF assessment of populations that include this vulnerable age is important. Additionally, it should be considered that many other factors reduce the possibility that people will be available for face-to-face testing. These include not only pandemics but also having debilitating illnesses that limit locomotion or the immune system, living in isolated locations, or others variables associated with poverty (e.g., shortage of means to pay for transport to and from research laboratories, or not having guardians who are available to accompany minors in person, which is often necessary). All of these conditions can also potentially impair EF, especially during sensitive phases of development like adolescence (Moffitt et al., 2011), which may go unnoticed if remote testing is not possible. Hunersen et al. (2021) call for the need to increase remote data collection strategies for testing adolescents, especially those from low-income settings using free tools, as proposed here.

The test battery used here was built for research purposes and group comparisons, not diagnostic evaluation, so norms are not available. For this reason, we matched participants tested online to others tested in person according to factors that are known to potentially affect brain and cognitive development (parental schooling, age, pubertal status, and sex: Foulkes and Blakemore, 2018). With the present study, we aim to establish whether applying the FREE battery is feasible under supervised online testing, to describe how the online testing was implemented and to compare the pattern of effects in both in-person and face-to-face conditions. Although completely remote, the supervised online presentation preserve some important aspects of in-person assessment that might be sources of bias if absent from online tests. These procedures included: (1) testee-examiner interaction throughout testing to prevent distraction from task objectives and misinterpreted instructions (Feenstra et al., 2017); (2) same format of stimuli presentation (PDF viewing of instructions and stimuli) that are seen (shared screen when online) concomitantly by the testees and examiner; (3) same type of response to the tests (vocal, in which the examiner writes down the responses); and (4) self-paced format (clicking a mouse or tapping a keyboard to progress to the next stimulus during online testing and swiping the screen for in-person testing); the examiner used a stopwatch to time how long testees took to complete each task and wrote down the answers. Because the procedures were essentially the same except for being administered in person or online, we hypothesized that performance would be equivalent.

## Materials and Methods

### Participants

Our convenience sample consisted of 96 native Portuguese-speaking, typically developing adolescents aged 9–16, of whom 48 were tested online. These individuals were matched to 48 adolescents who were evaluated in person at their schools (see matched pairing details below). Exclusion criteria were: (1) having been held back for a year or more at school; (2) being a student with special needs, which may be associated with clinical or cognitive limitations; and (3) taking daily medication to exclude any presence of chronic clinical disorders that could affect cognitive and/or developmental outcomes.

### Procedures

This study was approved by the local Ethics Committee (CAAE # 56284216.7.0000.5505). Prior to the testing sessions, participants’ guardians provided signed informed consent. Informed participant assent was always confirmed before test administration. Participants answered a socio-demographic questionnaire, self-evaluated their puberal status filling in the 5-item Pubertal Development Scale [PDS, adapted from Carskadon et al., 1993 into Portuguese by Pompéia, 2019] and were administered the FREE executive function tasks in four pseudorandom orders to avoid the effects of fatigue. Participants from the supervised online group (hereafter called as “online group”) were recruited through contact from their schools and social media after the authorities introduced social distancing and closed schools due to the COVID-19 pandemic. They were tested between 3 and 9months after lockdown. All online participants were individually paired/matched to in-person tested individuals recruited at their schools and assessed the year before the pandemic broke out (some of whom were part of a prior study: Zanini, 2021). Matching was based on sex, age, pubertal status, and parent’s average years of schooling as a proxy for cognitive stimulation, or socioeconomic status (SES; Sirin, 2005).

Both groups (online and in-person) were tested individually with supervision in a single session. Participants from the in-person group were tested at their schools using touchscreen tablets holding PDF files containing stimuli and instructions. Differently, participants tested online were at home and assessed remotely through an internet connection using their own hardware (computer or laptop, with web camara and basic free software such as Adobe Acrobat, PowerPoint, and a free Zoom video communication application). These individuals were instructed by the experimenter to share their screens (step-by-step written and oral instructions were provided for those unfamiliar with Zoom). Next, participants were helped to download tests in PDF format from their own e-mails or their guardian’s. These files were not available until testing started, so that they could not preview the tasks. For both groups, the examiner was present during the whole test session, supplying instructions, answering questions and ensuring participants were doing the tests as expected (e.g., not being interrupted by their cellphones and such like). All participants were awarded a “science partner” certificate after taking part and those tested in-person were reimbursed for their travel expenses. The EF test battery took around 40min to be completed, including instructions and rest breaks if the participants required them. Approximate time taken to complete each task was: 2min for both the Inhibition tasks, 4min to complete the Color Shape task and 6min to complete Category Switch (Shifting tasks), around 5min to complete 2-Back task and 8min to complete the Number Memory task (Updating tasks). Other tasks were administered to the same samples and their results will be reported elsewhere.

### Cognitive Measures

**Figure 1:**
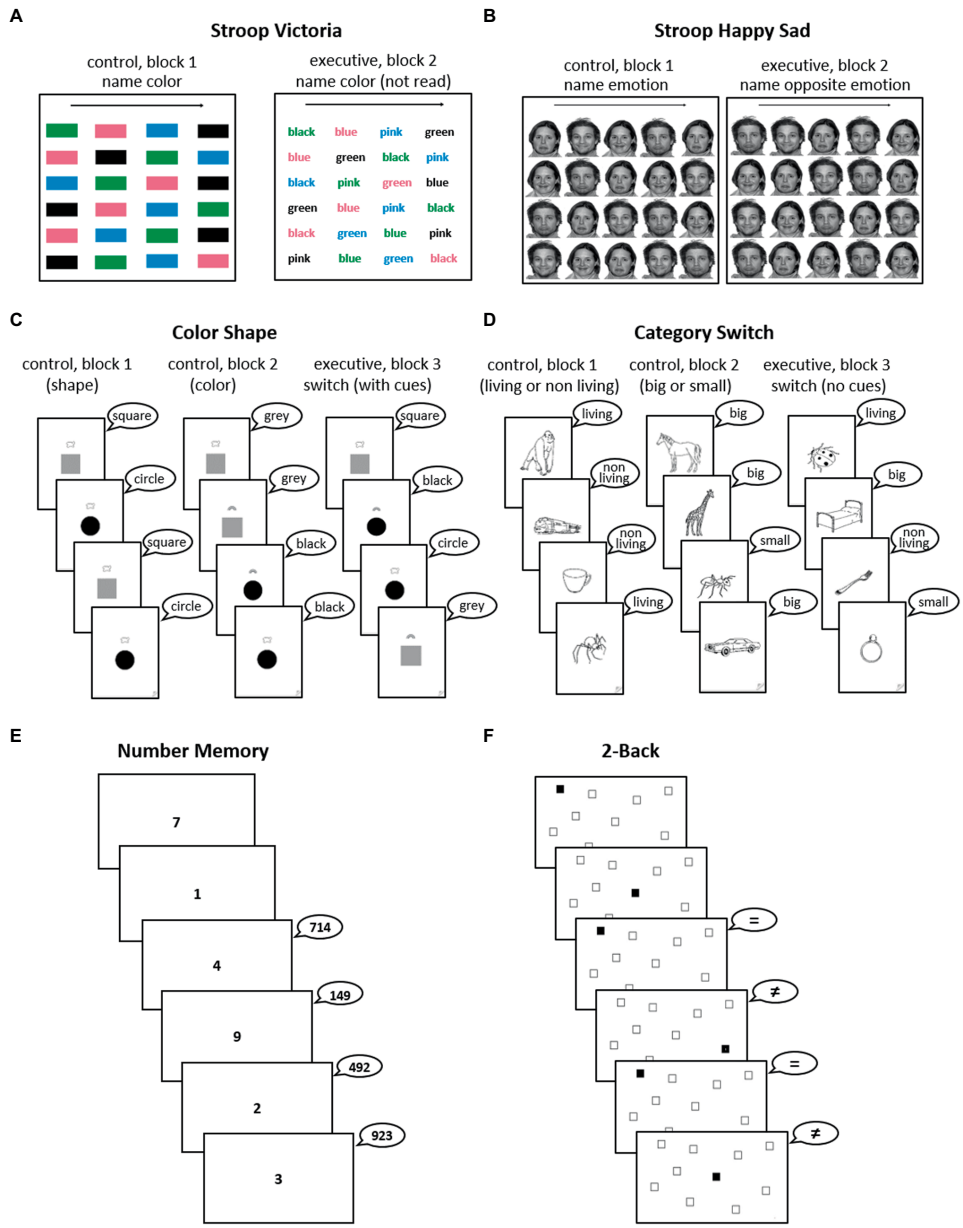
Overview of the two tasks of each of the three executive domains: inhibition tasks **(A,B)**, switching tasks **(C,D)** and updating tasks **(E,F)**. In the Inhibition and Switching tasks the first blocks are the control blocks (naming characteristics of stimuli, with no executive requirements) and the last block requires executive abilities in addition to those involved in the control blocks. For details, see Table 1 and Zanini (2021). All illustrated answers in speech bubbles are correct.

The FREE battery contains six tests adapted for use in diverse samples in terms of SES and cultural context. The theoretical basis for the battery, the rationale for choice of tasks, description of tasks, answer sheets and scoring method are detailed in Zanini (2021). A brief description of tasks and scoring procedure for each domain can be found in Table 1 and Figure 1. Following prior studies (see Zanini, 2021), the Inhibition and Switching tasks included blocks used to control for vocal/psychomotor speed and a corresponding block with the same requirements plus executive demands, while the Updating tasks contained no such control, as is the norm in this field.

**Table 1:** Description of the self-paced executive function tasks per domain and their corresponding scores (based on Zanini, 2021).

Domain (Task)	Paradigm	Scores
**Inhibition**		
(Stroop Victoria)	Contains two blocks, each of which consists of 24 stimuli (color patches or words) displayed on a single screen. Participants name the ink color of patches (block 1) and words that are color names written in incongruous ink colors (block 2). Block 1 is the control block, measuring speed to name colors. Block 2 involves inhibition (naming ink colors of words instead of reading the color names)	Cost of inhibition: RCS in block 2 minus RCS in block 1
(Happy-Sad Stroop)	Contains two scored blocks, each of which consists of 20 facial emotions that are displayed on a single screen. In block 1, participants name the emotions (happy or sad). In block 2, they inhibit naming the emotion and must name the opposite one (happy as sad or *vice-versa*)	Cost of inhibition: RCS in block 2 minus RCS in block 1
**Switching**		
(Color-Shape)	Contains three blocks in which single-colored geometric pictures are presented on each screen. As participants pass from screen to screen, pictures must be classified by shape (squares/circles) (block 1: 20 trials or screens), by color (black/gray) (block 2: 20 trials) or alternating (switching) classifications (block 3: 40 trials), according to cues presented above the pictures	Shifting costs: RCS in block 3 minus the sum of RCS in blocks 1 and 2
(Category Switch)	Contains three blocks in which single pictures are presented on each screen. As participant pass from screen to screen, each pictures must be classified as living or non-living (block 1: 20 trials or screens), big or small (block 2: 20 trial) or alternating (switching) classifications without cues (living/non-living, then big/small and so forth) (block 3: 40 trials)	Shifting costs: RCS in block 3 minus the sum of RCS in blocks 1 and 2
**Updating**		
(2-Back)	Each screen contains 10 square outlines in fixed locations, one of which is filled in with black ink. As participants pass from screen to screen, they answer if the black square location they see is in the same or a different location as the black square two screens back. The total number of updating opportunities is 66	Total RCS (for accuracy, in this case only: hits minus false alarms) (no control block)
(Number Memory)	Each screen contains a single digit number (1 to 9). As participants pass from screen to screen, they report the last three digits (trios) seen, in the same order as they were presented, having to continuously update information held in working memory, discarding the first digit of the trio and adding the new digit that appears next. The total number of updating opportunities is 24	Total RCS (no control block)

RCS=Rate Correct Score obtained by accuracy (vocal responses) divided by time (s) to complete blocks/task timed by the experimenter. See Figure 1 for a visual illustration of the task.

To carry out the executive function tasks, testees read the instructions or had them read to them if preferred. The examiner clarified any questions that came up. Answers were vocal and the tasks were self-paced (swiping to pass from instructions to stimuli and between stimuli for the in-person group and clicking a mouse or tapping a forward keyboard arrow in the online group). The examiner wrote down the vocal answers and timed testees’ task completions using a handheld stopwatch, akin to classic tests used to assess intelligence, for instance, following the long tradition of paper-and-pencil testing (for a further discussion on the advantages of this, see Kessels, 2019; Zanini, 2021). Sessions were recorded with participants’ and their guardians’ consent and erased once adequate scoring was ensured.

For each task or task block (depending on the test), the Rate Correct Score (RCS; see Vandierendonck, 2017) was calculated by dividing the number of correct answers by the time (in seconds) taken to complete each block. This metric controls for speed-accuracy trade-offs, that is, the between-participant variability in deciding to do tasks slowly, which can increase accuracy, or quickly, with higher error rates.

### Statistical Analyses

Descriptive and inferential analyses were conducted using IBM SPSS Statistics version 21 software. The scores entered as dependent variables in the statistical analyses of the Inhibition and Shifting tasks were the executive costs (RCS from the block with executive requirements minus the RCS from control blocks). These dependent variables for each task were used in separate univariate General Linear Models (GLMs) with the factor group (online vs. in-person). For the Updating^1^ measures, total RCS was used as the dependent variables in similar GLMs because they do not include baseline/control blocks. To correct for speed (of vocal responses and/or passing from stimulus to stimulus), our analyses included another continuous predictor, the mean RCS of the control blocks of the Inhibition and Shifting tasks, which have no executive requirements and basically involve answering aloud about an attribute of the stimuli (retrieval of phonological representations from long-term memory) and speed of progressing through all stimuli, which is also done in the Updating task (see Zanini, 2021).

Because there is no prior data on online administration of the FREE test battery in the literature, calculating sample sizes was not strictly possible. We therefore focused on determining effect sizes (unstandardized and standardized) of the effect of group, which are useful to indicate the magnitude of the reported effects in metrics that are comparable across the tests. Standardized effect sizes are also useful to communicate the practical significance of the results, for meta-analysis and, importantly, can be used in future studies to determine sample size if these investigations intend to compare participants tested online and in-person (see Lakens, 2013). Effect sizes were presented as partial eta squared provided by SPSS and Hedges g [using Excel spread sheet],^2^ with corresponding confidence intervals. Rules of thumb describe medium effect sizes as those, respectively, between 0.059–0.138 and 0.5–0.8; values equal to or higher than 0.138 and 0.8 are considered large effect sizes (see Richardson, 2011; Lakens, 2013). Because participants for the different groups were matched by sex, age, pubertal stage, and SES, these variables were not supposed to be different between groups, so we did not include them as covariates in the analyses. Data were inspected for outliers (values over three SD of the mean).

## Results

After matching participants tested online and in person (see Table 1), the proportion of participants of each sex, their age, and pubertal status and their parents’ average years of schooling were not different across groups. However, for one pair of matched participants there was a difference of 7years in the mean of parental schooling. We therefore sought to further control possible differences in cognitive stimulation between groups. As the in-person sample was mostly from public schools, except one, while all but one member of the online group were from private schools, the type of school (public vs. private) was included as a covariant in the analyses. This was done because Brazilian private-school students often outperform those from public schools (which tend to have lower quality education) on executive function tasks (e.g., Guerra et al., 2021).

The following outliers were found per variable: one in the Happy-Sad Stroop and one in the 2-Back for the in-person group and one in the Number Memory task for the online group. Both exclusion of these values or replacement for the value of the mean plus three SD retrieved similar results, so we report the results including data of these outliers.

**Figure 2:**
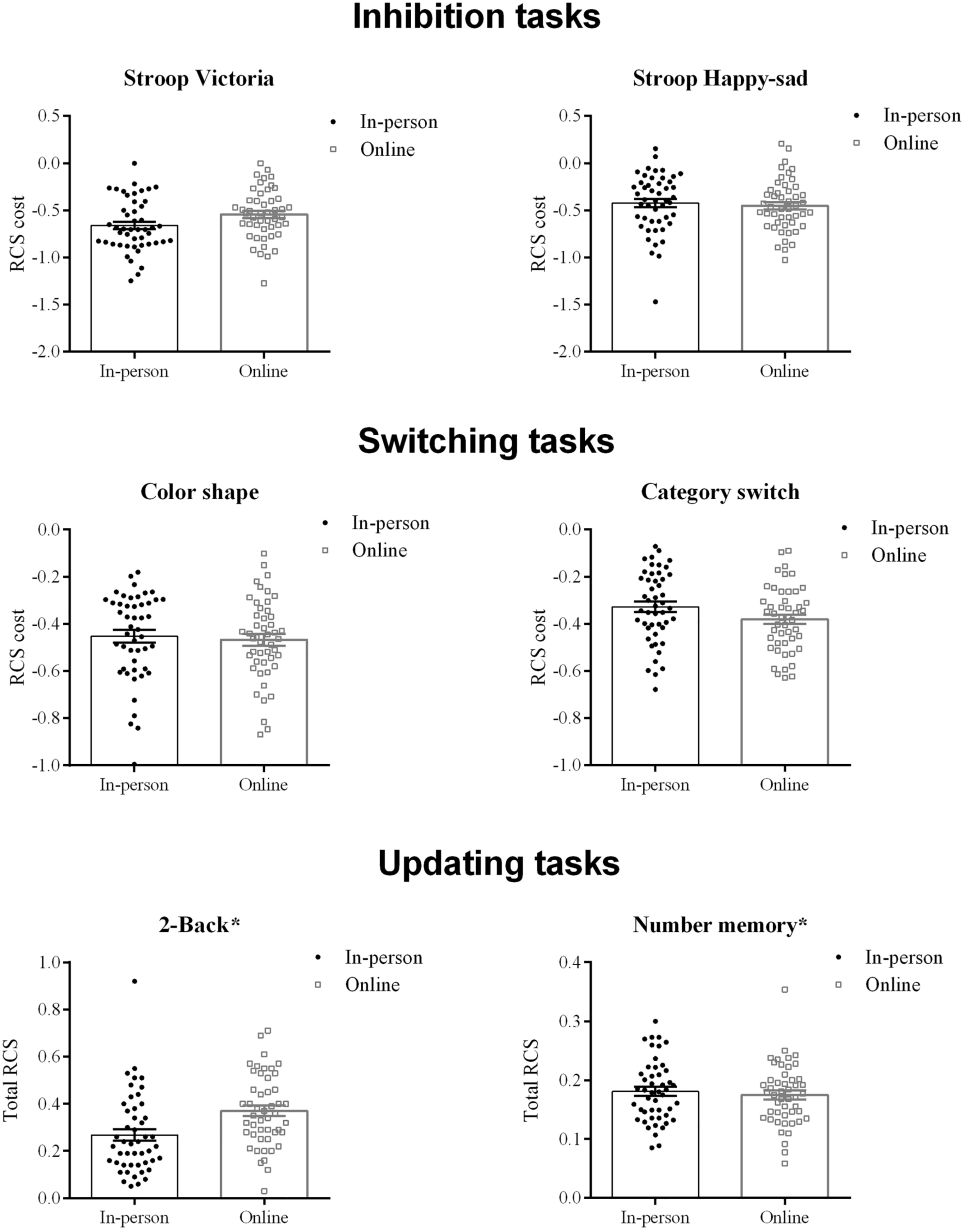
Individual (dots) and mean (±SE) scores (histograms with error bars) per type of test administration [participants tested in person (black circles) and online (open grey squares)] in each of the executive function measures. ^*^data shown without correction for speed.

Regarding type of test administration, we found no significant group effects (online vs. in person) in any of the executive tasks except for a marginal effect (small effect size) in the 2-Back test [*F*(1,92)=3.899, *p*=0.051, *p*_η_^2^=0.04], with a non-significant tendency for lower scores in the in-person group. No effects of type of school in any of the tasks were observed (see Table 2 and Figure 2), but there was an effect of the variable used to control for vocal/psychomotor speed in the 2-Back task [*F*(1,92)=23.49, *p*<0.001, *p*_η_^2^=0.20] and in the Number Memory task [*F*(1,92)=55.09, *p*<0.001, *p*_η_^2^=0.37], as expected, because the metrics used in the analysis of these tests do not have a baseline condition, unlike the inhibition and shifting tasks. The databank is available at https://osf.io/h5akr/?view_only=ea08777d698c46b4ae8110b9f8df8057t.

**Table 2:** Descriptive statistics of demographics and Rate Correct Scores (RCS: accuracy divided by total time in s) of executive functions performance.

Variables	In person (n=48; 21 girls)	Online (n=48; 21 girls)	Hedges g (95% CI)	Group effects			Type of School effects		
	Mean (±SD)	Mean (±SD)		F	p	p_η_^2^	F	p	p_η_^2^
**Demographics**
Age (years)	12.29 (1.97)	12.17 (1.96)							
PDS (score)	2.37 (0.71)	2.27 (0.71)							
Guardian’s schooling ($\overset{Â¯}{x}$ yrs.)	14.78 (2.27)	14.86 (2.58)							
**Executive functionsInhibition**
Stroop Victoria (inhibition cost)	−0.66 (0.28)	−0.54 (0.27)	−0.43 (−0.84/−0.03)	2.239	0.14	0.02	0.016	0.9	<0.01
Stroop Happy-sad (inhibition cost)	−0.44 (0.29)	−0.45 (0.27)	0.04 (−0.36/0.44)	0.878	0.88	0.01	0.728	0.40	0.01
**Switching**
Color-Shape (switching cost)	−0.45 (0.18)	−0.47 (0.17)	0.11 (−0.29/0.51)	0.028	0.87	0.001	0.038	0.85	<0.01
Category Switch (switching cost)	−0.33 (0.15)	−0.38 (0.14)	0.34 (−0.06/0.74)	1.525	0.22	0.02	0.009	0.93	<0.01
Updating (corrected for baseline speed)^*^
2-Back	0.27 (0.17)	0.37 (0.15)	−0.62 (−1.03/−0.21)	3.899	0.051	0.04	0.147	0.70	<0.01
Number Memory	0.18 (0.05)	0.17 (0.05)	0.20 (−0.20/0.60)	0.884	0.35	0.01	0.000	0.99	<0.01

PDS=Pubertal Development Scale; ^*^baseline speed effect for the 2-Back F(1,92)=23.49, p<0.001, p_η_^2^=0.20; Number Memory F(1,92)=55.09, p<0.001, p_η_^2^=0.37.

## Discussion

This study found no evidence that online, examiner-moderated use of the FREE test battery differs from in-person testing (effect were not significant and of small effect sizes). This suggests that remote testing this way may be a comparable alternative when face-to-face assessment is not possible as found for other cognitive test batteries (Krantz and Dalal, 2000; McGraw et al., 2000; Nosek et al., 2002; Temple et al., 2010; Soto et al., 2011; Germine et al., 2012; Cullum et al., 2014). Hence, performing self-paced tasks and responding vocally, either personally or online, and using different hardware under these conditions did not affect results. This makes sense considering that the executive function variables were controlled for speed of vocal responses and passing from one stimulus to the next, irrespective of the conditions and equipment used by the participants: smaller screens and swiping to progress to the next stimuli during face-to-face assessment, or larger screens (laptops or personal computers) and mouse or key presses, remotely.

This absence of significant difference between in-person and remote testing may seem surprising considering that there can be minor delays and variation in precision when transmitting images, sounds and registering motor responses over the internet, although these seem to vary little across browsers, platforms, and operating systems (Anwyl-Irvine et al., 2020). The similar performances in the testing conditions used here may be explained by the FREE executive tests’ design and scoring system, which is similar to classic paper and pencil tests, that have been used for decades and have considerable advantages over automated ones (Kessels, 2019). Time taken for each vocal answer is not the focus of interest in this test battery. Instead, it takes RCS into account, which is response accuracy divided by self-paced time taken for each task throughout a series of trials. Thus, total time to complete a task is much longer than reaction time per trial, which varies by milliseconds when computed in automatized tasks and could be affected by online transmission lags. Additionally, small variations in reaction times of examiners, who mark the beginning and end of each block of trials, are comparable in the control and executive conditions/blocks, online and in person, and become irrelevant when compared to total time taken to complete each block/task. Although this presents some extra workload for examiners, it allows testing under a wider variety of conditions (Kessels, 2019). These tasks can therefore be used by examiners who do not have access to special hardware and software that automatically time responses for each trial and in private practices or poorly equipped laboratories, given that most of the world’s neuropsychologists cannot afford these gadgets and applications. The present study shows that this advantage extends to online testing, as long as it is mediated by an examiner. In effect, inconsistent results have been found when comparing performance in tasks that are self-administered (e.g., in hospital vs. online settings: Feenstra et al., 2017), possibly because cognitive testing often needs supervision. Mediated testing allows examiners to make sure that testees understand tasks, pay attention when doing them and do not engage in the use of strategies that may distort performance.

We did find, however, a marginal group effect in the 2-Back task, which should be addressed. We envisage two possible explanations for this: (1) it could have been a spurious effect; and (2) although we controlled for type of school, it is possible that this statistical adjustment did not correct for a putative advantage of the online group, which had access to better schooling, having all been from private schools except for one individual. Indeed, Guerra et al. (2021) have recently shown that children from private schools in Brazil had higher spans for spatial locations (however, of a small effect size), but that spatial updating task performance, adjusting for span, did not differ between public and privately schooled individuals. Consequently, possible slightly higher spans due to higher cognitive stimulation could have contributed to the 2-Back results in the online group, which was not assessed here because of the nature of our 2-Back task, which differs from the n-back test of Guerra et al. (2021). In line with this idea, our findings show that the marginal non-significant effect in the 2-Back task does not seem to be specific to updating-related executive functioning, because group effects in the other task of this domain (Number Memory) were nowhere near significant, nor close to a medium effect size. It is also of note that, despite the lack of norms, the marginally significant difference found between scores from the in-person and online groups in the 2-Back task seem to have been due to a lower performance of the in-person group (mean±SD 0.27±0.17) because the online group (0.37±0.15) presented results in this task that are very similar (0.35±0.13) to those found by Zanini (2021), in which all participants were tested in person. Indeed, overall, performance of the online sample was very similar to that obtained in a comparable population using the same battery in person: means obtained here were within mean±0.5 SD of Zanini (2021), which correspond to low effect size differences (Lakens, 2013).

The FREE test battery was designed with two tasks of three executive domains, so that consistency of effects between each pair of tests of each type can be used to ascertain the influence of various factors such as mode of testing (online vs. in-person), sex, SES, and so forth. Having two tasks of each domain also allows latent factors to be estimated, considering many multifactor configurations found for the Unity and Diversity Model of Executive Functions (see Karr, 2018). Unlike raw scores used here, latent factors capture the common variance in performance across different tasks, free of measurement errors (Brown et al., 2015). Therefore, specific cognitive requirements of a task that are not shared with the corresponding test of the same domain (such as spatial span in the 2-Back but not the Number Memory task) should not contribute to the latent factor. Our sample was too small to explore the latent nature and best model configuration of the executive functions’ Unity and Diversity model, and to obtain evidence of invariance (Meade and Bauer, 2007) across mode of testing (online vs. in-person), so this must be undertaken in future studies. Another advantage of having a couple of tests per domain is that researchers who intend to use only one task of each EF type can pick the one which they deem more adequate for their purposes, although the ideal is to obtain latent scores. This, however, is only possible which large samples.

Considering the present scenario, evidence is emerging that COVID-19 and similar infections may lead to cognitive problems (cognitive COVID) beyond the acute stage (Ritchie and Chan, 2021), so repeatedly assessing infected patients may become essential to understand possible long-term cognitive impact. This includes children and adolescents, some of whom present long-term COVID effects (see Hertting, 2021) and will need to be followed up. As we have shown here, this may be done remotely with supervision, enabling a greater number of patients to be assessed if they have internet access, some familiarity with digital tools, a computer or device that runs basic software, a web camera, and a reasonably sized screen. All tests used may be easily adapted for diverse populations and are affordable (see Zanini, 2021) for poorly funded researchers.

Additionally, a point to bear in mind is that online testing may pose technical issues such as unreliable connections or slow speeds. Testees may also not be comfortable with the technology involved or not have the hardware and an internet connection, which might be the case for those from low SES. Furthermore, examiners may fail to notice difficulties that are not clear through vocal communication when participants are unable or unwilling to turn on their cameras (common in youngsters: Castelli and Sarvary, 2021). Online testing also poses some ethical problems that must be minimized such as violating privacy. Nonetheless, despite these shortcomings, online testing probably reaches much larger numbers at low cost and will therefore probably become more prevalent in the post-pandemic period.

The main limitation of the present study is that participants in the in-person group were tested before the pandemic and those tested online were assessed during the pandemic, so this could have affected the results. Ideally, both groups should have been evaluated in parallel, but due to the pandemic this was not ethically acceptable. Nonetheless, the negative acute effects of the lockdown, which seem to be more severe (Creswell et al., 2021), were avoided, as we only tested participants from 3months after the beginning of the social distancing measures. Additionally, potential participants who were reported by guardians as not being healthy were not tested in either group. If the COVID-19 crisis had affected participants in ways that interfered with their EF, beyond those that were controlled for by matching participants (which are the known factors that influence these cognitive abilities: Foulkes and Blakemore, 2018), it would be expected that the online group perform worse, which was not observed. Various factors that could have changed due to the pandemic, such as increases in mental health problems, sedentarism, body weight, alterations in sleep patterns, and so forth are not usually considered at all in prior studies that investigated the same EF model as used here (e.g., Friedman et al., 2011, who used the same sample of twins in many publications). Hence, it would be unclear how or whether they could have affected results. Another limitation of our study includes its sample size. Because our aim was to test feasibility of supervised remote online testing using the FREE battery, we did not have a sample large enough to allow us to run confirmatory factor analysis of the Unity and Diversity model of executive functions (see Friedman and Miyake, 2017; Karr, 2018) and to perform invariance testing and other types of validation techniques to ensure that web-based assessment was tapping the same constructs as those measured face-to-face (see Germine et al., 2012). Matching participants not only by parental schooling but also the type of school would have also been ideal. We attempted to control for the latter statistically, but it might have not been an effective control as almost all participants in the online group were from private schools, which more readily agreed to help volunteers participants during school closure. We had little success accessing families through public schools because their staff were much more severely overburdened due to the pandemic and extremely low governmental funding to aid the transition to online teaching. Finally, unlike other similar studies that investigated the adequacy of remote cognitive assessment by controlling for individual differences in performance using within-participant designs (e.g., Cullum et al., 2014; Feenstra et al., 2017; Backx et al., 2020), we did not test the same participant face-to-face and online because test–retest reliability of executive functions is known not to be high (Karlsen et al., 2020) because testees develop strategies that minimize executive functioning. On the upside, this experiment used a sample from a developing nation, which is still rare in the international literature (Rad et al., 2018), especially regarding the adequacy of remote cognitive assessment.

Overall, although our findings cannot be generalized to samples from other cultures and age groups, we have shown that online testing using the FREE test battery is a potentially viable means of remotely assessing EF. Because this test battery is open access, adaptable to populations with different characteristics and remote testing was done using only free software, our results provide initial evidence for a much-needed remote way to assess adolescents at low costs, including those who are more vulnerable to factors that negatively affect developmental (Hunersen et al., 2021; Moffitt et al., 2011), including BAME and non-WEIRD populations, who are under-represented in the cognitive literature (see Henrich, 2010; Rad et al., 2018). We conclude that online testing the way it was administered here is feasible way of collecting data on EF, making this a potential alternative when face-to-face testing is not possible. Until more controlled experiments are conducted, it is advisable to either test all participants online or in person and not mix these conditions, which was not assessed here.

## Data Availability Statement

The datasets presented in this study can be found at: https://osf.io/h5akr/?view_only=ea08777d698c46b4ae8110b9f8df8057t.

## Ethics Statement

The studies involving human participants were reviewed and approved by the Comitê de Ética em Pesquisa of the Universidade Federal de São Paulo. Written informed consent to participate in this study was provided by the participants’ legal guardian/next of kin.

## Author Contributions

SP and IS: designing the study, planning of the study, data collection, data analysis, and interpretation of the data. SP: public responsibility for the content of the article. All authors contributed to the article and approved the submitted version.

## Funding

Fundação de Amparo à Pesquisa do Estado de São Paulo (FAPESP – Processes: due to fellowships to author IS 2019/19709–6 and SP 2016/14750–0), Coordenação de Aperfeiçoamento de Pessoal de Nível Superior (finance code 001), Conselho Nacional de Desenvolvimento Científico e Tecnológico (CNPq – #301899/2019–3 due to fellowships to author SP), and Associação Fundo de Incentivo à Pesquisa (AFIP).

## Conflict of Interest

The authors declare no commercial or financial relationships that could be construed as a potential conflict of interest.

## Publisher’s Note

All claims expressed in this article are solely those of the authors and do not necessarily represent those of their affiliated organizations, or those of the publisher, the editors and the reviewers. Any product that may be evaluated in this article, or claim that may be made by its manufacturer, is not guaranteed or endorsed by the publisher.

## Footnotes

^1^For the 2-Back task, because participants can adopt a strategy of guessing (without actually updating the content in their working memory) by responding that all spatial configurations are different from the one presented two trials back (only 36% of trials are the same), accuracy in this case was calculated as hits minus false alarms (Jaeggi et al., 2010). Guessing in the other tasks can be picked up by the examiner but this never occurred in our experience.

^2^ https://www.cem.org/effect-size-calculator
